# Comparison of the Performance of Phenotypic Methods for the Detection of Carbapenem-Resistant Enterobacteriaceae (CRE) in Clinical Practice

**DOI:** 10.3389/fcimb.2022.849564

**Published:** 2022-02-21

**Authors:** Zhijie Zhang, Dayan Wang, Yahui Li, Yong Liu, Xiaosong Qin

**Affiliations:** ^1^ Department of Laboratory Medicine, Shengjing Hospital of China Medical University, Shenyang, China; ^2^ Department of Laboratory Medicine, Tacheng Hospital of China Medical University, Tacheng, China; ^3^ Department of Laboratory Medicine, Cancer Hospital of Anshan, Anshan, China

**Keywords:** carbapenem-resistant Enterobacteriaceae, combined disk test, modified carbapenem inactivation method, EDTA-modified carbapenem inactivation method, NG-Test CARBA 5, color developing immunoassay

## Abstract

In order to investigate the diagnostic performance characteristics of four phenotypic assays in detecting carbapenem-resistant Enterobacteriaceae (CRE), we collected the CRE strains from infected patients. The results of carbapenemase gene detection, *bla*_KPC-2_, *bla*_OXA-23_, *bla*_NDM-1_, *bla*_NDM-4_, *bla*_NDM-5_, *bla*_IMP-4_, and *bla*_IMP-8_, were used as a standard to evaluate the performances of combined disk test (CDT), modified carbapenem inactivation method(mCIM)/EDTA-modified carbapenem inactivation method(eCIM), NG-Test CARBA 5 (CARBA), and color developing immunoassay (CDI). The compliance of phenotype results based on CDT, mCIM/eCIM, CARBA, and CDI with genetic detection results was 94% (231/247), 95% (235/247), 98% (242/247), and 99% (246/247), respectively. CDT demonstrated a low specificity for carbapenemase detection, low negative predictive value (NPV), and low sensitivity for metallo-β-lactamase (79%, 55%, and 88%, respectively); it also failed to accurately detect IMP. The mCIM/eCIM assay had serious problems in detecting OXA-23-like carbapenemases. The sensitivity and specificity of CARBA and CDI were higher than those of the first two methods. However, CARBA did not cover the detection of OXA-23, while CDI cannot detect IMP-8, resulting in low NPVs (70% and 88%, respectively). In conclusion, CARBA and CDI assays are highly accurate except individual rare genes and allow direct genotype detections. CDT and mCIM/eCIM assays are moderately accurate and can only distinguish serine-β-lactamases from metallo-β-lactamases. Laboratories should choose the appropriate method that meets their needs based on its characteristic.

## Introduction

Carbapenem-resistant Enterobacteriaceae (CRE) produce carbapenemases as their primary antimicrobial-resistance mechanism. Carbapenemases are β-lactamases that belong to different Amber classes (A, B, and D). Class A carbapenemases are serine β-lactamases, with *Klebsiella pneumoniae* carbapenemases (KPCs) being the most common. Class B carbapenemases are metallo-carbapenemases (also known as metallo-β-lactamases, MBL), with NDM, IMP, and VIM as common types. Class D is predominantly OXA-48-like serine β-lactamases (Stojanoski et al., 2021).

CRE are usually characterized by multiple antimicrobial resistance, resulting in very limited therapeutic options available in clinical practice. Recently developed β-lactamase inhibitors showed different inhibitory effects on different carbapenemases; avibactam (Yin et al., 2019), vaborbactam (Novelli et al., 2020), and relebactam (Tooke et al., 2019) have inhibitory effects on class A carbapenemases, but not on metallo-β-lactamases. On the other hand, taniborbactam (Hamrick et al., 2020; Wang et al., 2020) can inhibit class A, class B, and OXA-48-like carbapenemases in class D. The same antimicrobial agent has a slightly different susceptibility against different types of carbapenemase. For example, strains carrying class B and D beta-lactamases are susceptible to aztreonam, while strains producing class A carbapenemases are resistant. Given the distinctive features of different carbapenemases and the limitations of available antimicrobials, clinical laboratories are expected to have the ability to detect carbapenemase types. Especially for laboratories with limited antimicrobial susceptibility testing capabilities, the identification of carbapenemases would be an important guide for clinicians in selecting antimicrobials.

Recently, a lot of Phenotypic detection methods of CRE has been developed, such as Nitro Speed-CarbaNP Test (Nordmann et al., 2020), Rapidec CarbaNP (Elawady et al., 2019), CarbaNP-Direct test (Banu et al., 2019), MALDI-TOF MS rapid detection method (Oho et al., 2021), Fluorogenic Assay method (Kim et al., 2020) and so on. A large proportion of methods were very complex or need very expensive instrument and aren’t fit the use of common clinical laboratory.

Based on previous studies, two phenotypic assays that are easy to perform in clinical laboratories and two immunological enzyme detection reagents produced in China were selected for evaluation and comparison, using genetic detection results as the gold standard. Our aim was to provide informative references for laboratories to carry out carbapenemase assays.

## Materials and Methods

### Strains Sources

In total, 185 CRE strains (152 Klebsiella pneumoniae, 14 Escherichia coli, 10 Enterobacter cloacae, 4 Serratia marcescens, 3 Enterobacter aerogenes, 1 Citrobacter freundii, and 1 Proteus mirabilis) from patients at Shengjing Hospital of China Medical University from January 1, 2019, to June 30, 2021, and 62 CRE strains (45 K. pneumoniae and 17 E. coli) from 14 hospitals in China in 2020 were collected and included in this study.

The strains from the 14 hospitals were collected through a research project of the National Science and Technology Basic Research Program of China (2019FY101200). These hospitals include the Peking Union Medical College Hospital, Hebei Yanda Medical Research Institute, No. 3201 Hospital, Second Affiliated Hospital of Nanchang University, Tongji Hospital of Huazhong University of Science and Technology, Henan Provincial People’s Hospital, Xiangya Hospital of Central South University, First Affiliated Hospital of Guangzhou Medical University, People’s Hospital of Ningxia Hui Autonomous Region, Children’s Hospital of Soochow University, West China Hospital of Sichuan University, General Hospital of Tianjin Medical University, Beijing You’an Hospital, Yunnan Cancer Hospital.

Of the 197 K*. pneumoniae* strains included in this study, 32% (64/197), 28% (55/197), and 16% (31/197) were derived from qualified sputum, blood, and urine specimens, respectively. The largest proportion of the 31 *E. coli* strains originated from urine specimens, accounting for 45% (14/31), while blood and qualified sputum specimens accounted for 13% (4/31) and 6% (2/31), respectively.

### Strain Isolation and Identification

Specimens (blood or other local samples) were obtained from suspected infected patients. Different agar plate or broth (include blood culture bottles) and various environment were adopted for the culture of different specimens. Pure colonies of agar plate (bacterial in broth were subculture to agar plate regularly) were identified with VITEK-MS mass spectrometer (bioMerieux, France). Results with identification percentages ≥85% were considered reliable.

### Antimicrobial Susceptibility Tests

Strains identified as species of Enterobacteriaceae were checked for antimicrobial susceptibility to imipenem, meropenem, and ertapenem use disk diffusion method (CLSI M02-A12, 2015). Strains resistant to at least one of the three antimicrobials were included in this study. *E. coli* strain ATCC25922 and *Pseudomonas aeruginosa* strain ATCC27853 as the quality control strains. The results were interpreted according to the Clinical and Laboratory Standards Institute (CLSI) M100-S31 guidelines (CLSIM100-S31, 2021).

### Antimicrobial-Resistance Gene Detection

The enrolled strains were cultured overnight on Columbia blood agar plates. Pure colonies were selected for DNA extraction using a boiling method (suspension of bacterial were boiled in boiling water for 15min, rapid cooling in ice water, centrifuge 10000rpm for 10min,supernatant were taken out for use), and *bla*_KPC_, *bla*_NDM_, *bla*_IMP_, *bla*_VIM_, *bla*_OXA-48_, and *bla*_OXA-23_ genes were amplified. The detailed information of primers and amplification conditions (Banu et al., 2019) are listed in **Table 1**. Product of amplify were observed through agarose electrophoresis. The amplified gene fragments were sequenced and compared with Basic Local Alignment Search Tool (BLAST)to define specific genotypes.

**Table 1:** Primers and amplification conditions of common carbapenemases.

Primer	Sequence	Sequence length (bp)	Amplification condition
*bla*_KPC_	F-5’-ATGTCACTGTATCGCCGTCTA-3′ R-5’-TTACTGCCCSKTGACGCCCAA-3′	822	94°C for 5min, 30×(94°C for 60 s, 55°C for 45 s, and 72°C for 60 s), 72°C for 5 min
*bla*_NDM_	F-5’-GGTCGCGAAGCTGAGCACCGCAT-3′ R-5’-GCAGCTTGTCGGCCATGCGGGC-3′	782	94°C for 4 min, 30×(94°C for 30 s, 71°C for 30 s, and 72°C for 50s), 72°C for 10 min
*bla*_IMP_	F-5’-GARGGYGTTTATGTTCATAC-3′ R-5’-GTAMGTTTCAAGAGTGATRC-3′	587	94°C for 5 min, 30×(94°C for 60 s, 55°C for 45 s, and 72°C for 60s), 72°C for 5 min
*bla*_VIM_	F-5’-GTTTGGTCGCATATCKCAAC-3′ R-5’-AATGCGCAGCACCAGGATAG-3′	382	94°C for 4 min, 30×(94°C for 30 s, 58°C for 30 s, and 72°C for 40 s), 72°C for 10 min
*bla*_OXA-48_	F-5’-TTGGTGGCATCGATTATCGG-3’ R-5’-GAGCACTTCTTTTGTGATGGC -3’	744	95°C for 5 min, 30×(94°C for 1 min, 62°C for 1 min, and 72°C for 1 min), 72°C for 5 min.
*bla*_OXA-23_	F: 5’-TGTACGGTTCAGCATAATTTA-3’ R: 5’-AGATGCCGGCATTTCTGACCG-3’	699	95°C for 5 min, 30×(94°C for 1 min, 62°C for 1 min, and 72°C for 1 min), 72°C for 5 min.

### Combined Disk Test (CDT)

The strains to be tested were inoculated on Columbia blood agar plates and incubated overnight at 35°C. To prepare a 0.5 Mcfarland standard turbidity bacterial suspension, three to five pure colonies were taken. This suspension was spread on Mueller-Hinton (MH) agar plates, following the same protocol of previous disk diffusion tests. Four test disks containing meropenem marked with serial numbers 1, 2, 3, and 4 were applied to each plate. Then, 10 μL of phenylboronic acid (400 μg, 40 mg/mL) and 10 μL of EDTA (Ethylene Diamine Tetraacetic Acid,292 μg, 29.2 mg/mL) were added to disks #2 and #3, respectively; 10 μL each of phenylboronic acid and EDTA were added to disk #4. Then, all plates were incubated at 35°C for 18–24 h (Tsakris et al., 2010; Kumar et al., 2018).

**Figure 1:**
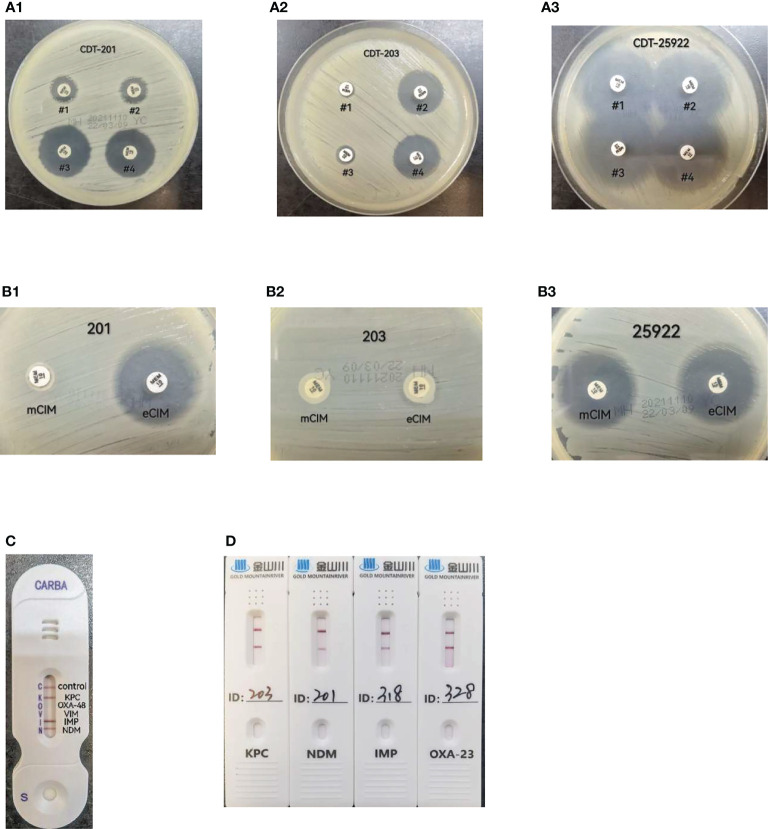
**(A)** CDT(combined-disk test): #1 meropenem only, # 2 meropenem+phenylboronic acid, #3 meropenem+EDTA, #4 meropenem+phenylboronic acid+ EDTA. A-1, strain 201, metallo-carbapenemases positive result; A-2, strain 203, serine-carbapenemases positive result; A-3, ATCC25922,negative result; **(B)** mCIM/eCIM. B-1, strain 201, metallo-carbapenemases positive result; B-2, strain 203, serine-carbapenemases positive result; B-3, ATCC25922,negative result; **(C)** CARBA-NG5 results for the KPC,IMP and NDM carbapenemases detected, **(D)** CDI result for strains 203,201,318,328 produce KPC,NDM,IMP and OXA-23 carbapenemases respectively.

Results observation: If the inhibition zones around disk #2 and #4 increased in diameter by ≥5 mm compared to those around disk #1, the strain was considered to be positive for class A or class C serine-β-lactamase production. If the inhibition zones around disk #3 and #4 increased in diameter by ≥5 mm compared to those around disk #1, the strain was considered to be positive for metallo-β-lactamase production. Either of the two cases was recorded as positive for carbapenemase production or negative if neither occurred (Tsakris et al., 2010; Kumar et al., 2018) (**Figure 1A**).

### Modified Carbapenem Inactivation Method (mCIM) and EDTA-Modified Carbapenem Inactivation Method (eCIM) Tests

All tests and result interpretation were performed according to the requirements shown in Table 3C of CLSI-M31 (CLSI M100-S31, 2021) (**Figure 1B**).

### Immunochromatographic Assay NG-Test CARBA 5 (CARBA)

For this test, 1 μL of fresh bacteria cultured overnight was added to 150 μL of extraction buffer. This was vortexed for a few seconds to make a homogeneous mixture and left at 20-25°C for 10 min. About 100 μL of the prepared mixture was added in a sample well of a test card with a disposable pipette, and the result was read after 15 min and interpreted according to test kit instructions as follows. If only one line appeared in the control region (C), the result was interpreted as negative. If the control line (C) did not appear, it meant that the reagent was out of control and the test was invalid. If one line in the control region (C) and one or several lines in the test regions K, O, V, I, and N appeared, the result was interpreted as positive, indicating that the sample contained KPC, OXA-48, VIM, IMP, and NDM-type carbapenemases, respectively (Bouta et al., 2018, manufacturer’s manual). The NG-Test CARBA 5 kits used in this study were produced by Changsha Zhong Sheng Zhong Jie Biotechnology Co. (**Figure 1C**)

### Color Developing Immunoassay Test (CDI)

Bacterial colonies were suspended in an EP(Eppendorf) tube containing 10 drops of sample treatment solution. This was mixed thoroughly with a disposable loop; then, 50 μL of the mixture was added in a sample well of a test card, and the result was read at 10 min. KPC, NDM, IMP, VIM, OXA-48-like, and OXA-23-like carbapenemases were detected with different test cards. The test results were read and interpreted as follows. The presence of both test and control bands was interpreted as a positive result. The presence of one control band only was interpreted as a negative result, while the absence of a control band meant that the reagent was invalid (manufacturer’s manual). The CDI test kits used in this study were produced by Gold Mountainriver (Beijing) Technology Development Co. (**Figure 1D**).

### Quality Control of Four Methods

*E. coli* strain ATCC25922 was used as a negative control, strain #203 and #201 in this study were identified *K.pneumoniae* and carry KPC-2 and NDM-5 gene respectively by sequencing method. They were used as a positive control for serine β-lactamase production and metallo-β-lactamase production.

### Statistical Analysis

The compliance, sensitivity, specificity, positive predictive value (PPV), and negative predictive value (NPV) of the four phenotypic assays compared with genetic detection results were calculated.

## Results

### Carbapenemase Gene Detection Results

As shown in **Table 2**, 233 (94%) of 247 CRE strains produced carbapenemases. The major carbapenemase-encoding genes carried were *bla*_KPC_ (154 strains, from 153*K. pneumoniae* and 1*E. coli*) and *bla*_NDM_ (66 strains, from 34*K. pneumoniae*, 24*E. coli* and 8 other bacterial). All the genes encoding *bla*_KPC_ identified in this study were *bla*_KPC-2_, with *bla*NDM-5 being the most common NDM gene. No *bla*_VIM_ or *bla*_OXA-48_ were identified. Six *Enterobacter cloacae* strains and two *K. pneumoniae* strains were found positive for *bla*_IMP_, all of which were *bla*_IMP-4_, except for strain 323, which had *bla*_IMP-8_. *bla*_OXA-23_ was detected in two *Serratia marcescens* strains, one *Enterobacter aerogenes* strain, one *E. coli* strains, and one *K. pneumoniae* strain.

**Table 2:** Types of carbapenemases gene in various species of Enterobacteriaceae.

Bacterial (No)	Result of carbapenemases gene (n, %)						
	KPC-2	OXA-23	NDM-5	NDM-4	NDM-1	IMP	No detect
*K. pneumoniae (*197)	153	1	24	1	9	2	8
*E. coli *(31)		1	20		4		6
*E. cloacae* (10)	1		2		1	6	
*E. aerogenes* (3)		1			1		1
*S.marcescens* (4)		2	2				
*C. ferrodii* (1)					1		
*P.mirabilis* (1)					1		
Total (247)	154 (62)	5 (2)	48 (19)	1 (0)	17 (7)	8 (3)	14 (6)

### Comparison of the Results of the Four Phenotypic Assays

The results of CDT, mCIM/eCIM, CARBA, and CDI methods for detecting antimicrobial-resistance phenotypes in 247 CRE strains showed 94% (231/247), 95% (235/247), 98% (242/247), and 99% (246/247) compliance with the genetic detection results, respectively (detailed test results are shown in **Table 3**).

**Table 3:** Results of four phenotypic detection methods for CRE.

Gene	Bacterial	No	CDT				mCIM/eCIM				CARBA					CDI				
			Ser	Met	Ser + Met	Neg	Ser	Met	Neg	Uncert	KPC	OXA-23	NDM	IMP	Neg	KPC	OXA-23	NDM	IMP	Neg
**KPC**	*K. pneumoniae*	153	153				152	1			153	No detect				153				
	*E. coli*	0																		
	Others	1	1				1				1					1				
**OXA-23**	*K. pneumoniae*	1				1			1						1		1			
	*E. coli*	1	1						1						1		1			
	Others	3	3						3						3		3			
**NDM**	*K. pneumoniae*	34		33	1			34					34					34		
	*E. coli*	24		22	1	1	2	22					24					24		
	Others	8	1	6		1	1	7			1*		7*		1	1**		7**		1
**IMP**	*K. pneumoniae*	2				2			2					2					2	
	*E. coli*	0												0					0	
	Others	6		1	1	4		6						6					5	1
**No detect**	*K. pneumoniae*	7	1	1		5		1	5	1					7					7
	*E. coli*	6				6			6						6					6
	Others	1	1						1						1					1
**Total**		247	161	63	3	20	156	71	19	1	154		65	8	20	154	5	65	7	16

Ser, serine-β-lactamase;Met,metallo-β-lactamase;Neg,negative;uncert,uncertain;CDT, Combined disk test;mCIM,Modified carbapenem inactivation method; eCIM,EDTA-modified carbapenem inactivation method;CARBA,Immunochromatographic assay NG-Test;CDI,Color developing immunoassay test;*KPC and NDM all positive of CARBA test in 1 S.marcescens. ** KPC and NDM all positive of CDI test in 1 S.marcescens.

The sensitivity, specificity, PPV, and NPV statistics of each assay are shown in **Table 4**. CDT had poor performances in terms of specificity (79%) and NPV (55%) for carbapenemase detection and sensitivity (88%) to metallo-β-lactamase detection, while the rest was above 93%. This low specificity for carbapenemase detection was associated with false-positive results in three cases of non-carbapenemase-producing strains. This low NPV was associated with the high number of false-negative results; six of the nine strains that yielded false-negative results produced IMP, accounting for 75% (6/8) of all IMP-producing strains. Similarly, the low sensitivity of CDT for metallo-β-lactamase detection was associated with a failure to detect IMP.

**Table 4:** Evaluation for the result of four phenotypic detection methods.

Test method	Result	Evaluation of result							
		TP	FP	TN	FN	Sensitivity (%)	Specificity (%)	PPV (%)	NPV (%)
**CDT**	carbapenemase	224	3	11	9	96	79	99	55
	serine-β-lactamase	158	6	82	1	99	93	96	99
	metallo-β-lactamase	65	1	172	9	88	99	98	95
**mCIM/eCIM**	carbapenemase	226	1	13	7	97	93	100	65
	serine-β-lactamase	153	3	85	6	96	97	98	93
	metallo-β-lactamase	69	2	171	5	93	99	97	97
**CARBA**	KPC	154	1	92	0	100	99	99	100
	NDM	65	0	181	1	98	100	100	99
	IMP	8	0	239	0	100	100	100	100
	Total	227	0	14	6	97	100	100	70
**CDI**	KPC	154	1	93	0	100	99	99	100
	OXA-23	5	0	242	0	100	100	100	100
	NDM	65	0	181	1	98	100	100	99
	IMP	7	0	239	1	88	100	100	100
	Total	231	0	14	2	99	100	100	88

TP, ture positive;FP, false positive;TN, ture negative;FN, false negative;PPV, positive predictive value;NPV, negative predictive value. CDT, Combined disk test;mCIM, Modified carbapenem inactivation method; eCIM, EDTA-modified carbapenem inactivation method;CARBA, Immunochromatographic assay NG-Test;CDI, Color developing immunoassay test.

mCIM/eCIM only scored low in NPV (65%) for carbapenemase detection, with the rest were above 93% in all metrics. This low NPV was associated with false-negative results for seven strains, of which five produced OXA-23-like carbapenemases. mCIM/eCIM assay was unable to detect them accurately.

CARBA tests excelled in terms of accuracy in carbapenemase detection, with all indicators above 97% and several others reaching 100%, except for a low NPV of 70% for all genotypes. This low NPV was caused by the fact that the reagent did not cover the detection of OXA-23-like carbapenemase. CDI had statistics above 98% for most indicators in carbapenemase detection, except for its sensitivity to IMP and NPV for all carbapenemase genotypes, which were 88%. The reason for this was that the IMP-8 producing strain 323 failed to be detected by CDI.

Both CARBA and CDI failed to detect the NDM-1 carbapenemases produced by the only *P.mirabilis* strain in this study. In contrast, for one *S. marcescens* strain that produced NDM-1 carbapenemase only, CARBA and CDI tests showed positive results for both NDM and KPC.

The assessment results for the ease of operation of all four assays are shown in **Table 5**. CDT and mCIM/eCIM required additional preparation of reagents, but their protocols were not complicated, and the reagents were of long validity. CDT and mCIM/eCIM took approximately 24 h to deliver results, whereas CARBA and CDI took only up to 30 min. However, CARBA and CDI reagents were expensive, costing 150–195 China Yuan (CNY), while CDT and mCIM/eCIM reagents only cost 10–15 CNY.

**Table 5:** Evaluation the ease of performance of four methods.

Test method	Clasification of carbapenemase	Preparation of reagent	Reagent storage environment and time	report time	Handing time (each strain)	Manualoperation times	Cost(CNY)
**CDT**	Main categories	2(phenylboronic acid,EDTA)	20-25°C, half to one year	18-24hours	3-5min	2	10
**mCIM/eCIM**	Main categories	1(EDTA)	20-25°C, half to one year	22-28hours	3-5 min	3	15
**CARBA**	Detailed classification	No	20-25°C, 1 year	25-30 min	3-5 min	2	195
**CDI**	Detailed classification	No	20-25°C, 2 year	10-15 min	3-5 min	1	150

CDT, Combined disk test;mCIM,Modified carbapenem inactivation method; eCIM,EDTA-modified carbapenem inactivation method;CARBA,Immunochromatographic assay NG-Test;CDI,Color developing immunoassay test;CNY,china Yuan.

## Discussion

There are two main problems with the CDT method. The first is the issue of false-positive results; 3 of the 14 non-carbapenemase-producing strains were misclassified as positive for serine-β-lactamases or metallo-β-lactamases. This led to a reduction in the specificity for carbapenemase detection to 79%. Phenylboronic acid only inhibits class A or class C serine beta-lactamases production and does not inhibit class D beta-lactamases (Sgrignani et al., 2016). So, CDT will fail to accurately pick up class D beta-lactamases. If a strain overexpresses a class C beta-lactamase and also has loss of porins or increased efflux it may test as a CRE (Fujiu et al., 2021) and also be false positive on CDT with phenylboronic acid. The other probably for false positives on CDT is the limitation to which genes were detected *via* PCR/sequencing. Other rarer carbapenemases (SME, NMC, GES) may also be present in these strains leading to false positive (Bonnin et al., 2020).

The second problem is its inability to accurately detect IMP-type carbapenemases which result in an NPV of only 55% for carbapenemase detection and a reduced sensitivity of 88% for metallo-β-lactamase detection. Undetected of IMP-type carbapenemases and individual false negatives for NDM carbapenemases, may be related to the inadequate expression of gene. The CDT only works if the MBL is well expressed and inhibited by EDTA.

In the study results of Tsakris et al. (2010), the sensitivity and specificity of CDT for detecting KPC were 100% and 98.8%, which are higher than the 99% and 93% in the present study, respectively. In contrast, according to Kumar et al. (2018), the detection rate of KPC by CDT was only 66.7%. However, considering that there were only 12 KPC-producing strains in this study, the possibility of data bias cannot be excluded. Together with the potential differences in strains from different geographical regions, this may also be one of the reasons for the different study results. In a study by Franklin et al. (2006), the focus was on the detection ability of the CDT method for metallo-β-lactamases. It was concluded that the detection rate of IMP-4 type metallo-β-lactamases by CDT was 100% in Enterobacteriaceae bacteria, which is significantly different from our results. In examining the details of their test protocol, it was found that Franklin et al. used imipenem disks, and the criteria for determining a positive result was >4 mm; both are different from our study (meropenem disks and ≥5mm). For Enterobacteriaceae that produce OXA-48-like carbapenemases, the sensitivity of the CDT method was only 58.5% (Hojabri et al., 2019). No strains producing OXA-48-like carbapenemases were found in our study, but for the five strains producing OXA-23-like carbapenemases, the detection rate was 80% (4/5). Despite both OXA-48 and OXA-23 are class D carbapenemases, the sensitivity of CDT for their detection may still be greatly different, and the two cannot be considered the same.

Studies have shown that the sensitivity and specificity of mCIM for carbapenemase-producing Enterobacteriaceae detection were generally above 97% (Lutgring et al., 2018; Sfeir et al., 2019; Zhong et al., 2020) and up to 100% in some cases (Tsai et al., 2020; Kumudunie et al., 2021), including that for strains producing OXA-48-like carbapenemases (Kumudunie et al., 2021). The 97% sensitivity and 93% specificity attained by mCIM in this study also confirmed it as a comparatively accurate phenotypic detection method. However, the fact that none of the five OXA-23-positive strains in this study was detected by mCIM should be considered. The significance of mCIM should be re-evaluated in hospitals or areas where OXA-23-like carbapenemase-producing strains are prevalent. In this study, the sensitivity and specificity of the eCIM test for metallo-β-lactamase detection were 93% and 99%, respectively, and 3 out of 63 (5%) NDM-producing strains were detected as serine β-lactamase, and two out of eight (25%) IMP-producing strains were detected as carbapenemase-negative by eCIM. These results are comparable to those of Tsai et al. (Tsai et al., 2020) (sensitivity of 89.3% and specificity of 98.7%). However, a study on *P. aeruginosa* (Gill et al., 2020) argued that the eCIM test could only accurately recognize NDM and VIM producers, but it was completely unable to detect IMP and SPM, which disagrees with our study. This suggests that the accuracy of phenotypic tests is not only related to antimicrobial-resistance genes but may also be related to bacterial species and other characteristics. Therefore, it is important for each locality to validate the testing of local strains to learn their specific accuracies.

The commercial reagents CARBA and CDI showed a high compliance with genetic detection results (98% and 99%, respectively), which is consistent with those in other studies (>92%) (Hopkins et al., 2018; Takissian et al., 2019; Liu et al., 2021). But, because of the characteristic of method of the CARBA and CDI (multiplex immunochromatographic assays, that is antibody based detection of expressed proteins), if the beta-lactamase isn’t expressed well or if the beta-lactamase has amino acid substitutions at the main epitope of the antibody, it may be false negative.

The common defect of the two reagents CARBA and CDI lies in the deviations of the detection results of one *P. mirabilis* strain and one *S. marcescens* strain, the specific reason of which needs to be further investigated. It is relatively clear that the CARBA reagent did not cover the detection of OXA-23-like carbapenemases. Therefore, its sensitivity will be significantly reduced when applied in regions with a high OXA-23 prevalence. Of the five OXA-23 producers in this study, four (*E. coli*, *Enterobacter aerogenes*, and *S. marcescens*) were from Shengjing Hospital of China Medical University. This serves as a reminder that Shengjing Hospital needs to be alert to the problem of missed detection of OXA-23 when using CARBA reagents for enzyme-based testing. The detection of IMP by CDI did not cover IMP-8, which is a deficiency compared with CARBA, and areas with a high IMP-8 prevalence should apply CDI in a prudent manner.

In summary, the four carbapenemase phenotypic methods validated in this study are among the many hotspot methods evaluated, and these are relatively easy to apply in clinical laboratories. Each of them has its own pros and cons. CARBA and CDI are highly accurate (except for CARBA did not cover the detection of OXA-23, while CDI cannot detect IMP-8), easy to perform, have a short turnaround time, and allow direct genotype detections, but these are also expensive and require a private payment of patients. CDT and mCIM/eCIM assays are moderately accurate, slightly cumbersome (requiring additional reagent preparation), time-consuming, and can only differentiate serine-β-lactamase/metallo-β-lactamase; however, these are inexpensive and do not require additional charges from patients. Therefore, each laboratory should pick the appropriate phenotypic assay according to its local and own specific circumstances.

## Data Availability Statement

The raw data supporting the conclusions of this article will be made available by the authors, without undue reservation.

## Ethics Statement

The samples were collected during routine care. Ethical review and approval was not required for the study of human participants in accordance with the local legislation and institutional requirements. Written informed consent from the patients or patients legal guardian was not required to participate in this study in accordance with the national legislation and the institutional requirements.

## Author Contributions

ZZ performed the analysis and wrote the manuscript. DW and YaL performed the portion test with ZZ. YoL implemented and coordinated the research. XQ designed and conceptualized the study. All authors contributed to the article and approved the final version.

## Funding

This research was funded by the Natural Science Foundation of Liaoning Province (project code: 2020-MS-10), Special Foundation for National Science and Technology Basic Research Program of China, Grant/Award Number: 2019 FY101200.

## Conflict of Interest

The authors declare that the research was conducted in the absence of any commercial or financial relationships that could be construed as a potential conflict of interest.

## Publisher’s Note

All claims expressed in this article are solely those of the authors and do not necessarily represent those of their affiliated organizations, or those of the publisher, the editors and the reviewers. Any product that may be evaluated in this article, or claim that may be made by its manufacturer, is not guaranteed or endorsed by the publisher.
